# Characteristics and antithrombotic treatment patterns of patients with concomitant coronary artery disease and atrial fibrillation from Thailand’s COOL-AF registry

**DOI:** 10.1186/s12872-021-01928-4

**Published:** 2021-03-02

**Authors:** Arjbordin Winijkul, Pontawee Kaewkumdee, Ahthit Yindeengam, Rungroj Krittayaphong

**Affiliations:** Division of Cardiology, Department of Medicine, Faculty of Medicine Siriraj Hospital, Mahidol University, 2 Wanglang Road, Bangkoknoi, Bangkok, 10700 Thailand

**Keywords:** Atrial fibrillation, Coronary artery disease, Multicenter registry, Anticoagulant, Antiplatelet

## Abstract

**Background:**

Concomitant coronary artery disease (CAD) and atrial fibrillation (AF) are common in clinical practice. The aim of this study was to investigate the characteristics and antithrombotic treatment patterns of patients with concomitant CAD and AF from the COhort of antithrombotic use and Optimal INR Level in patients with non-valvular atrial fibrillation in Thailand (COOL-AF Thailand) registry.

**Methods:**

Registry enrollment criteria included patients aged ≥ 18 years who were diagnosed with AF for any duration at any of 27 public hospitals located across Thailand during 2014–2017. The That Clinical Trials Registry study registration number is TCTR20160113002. Statistical comparisons of characteristics and treatment strategies were performed between patients with and without CAD.

**Results:**

Of a total of 3461 AF patients, 557 had concomitant CAD (16.1%). Patients with concomitant CAD and AF were significantly older, more likely to be male, had more comorbidities, and had more cardiovascular implantable electronic devices. History of stroke/transient ischemic attack and prior bleeding was not significantly different between groups. CHA_2_DS_2_-VASc score and HAS-BLED score were both higher in patients with CAD than in patients without CAD (4.17 *vs.* 2.78, *p* < 0.001, and 2.01 *vs.* 1.45, *p* < 0.001, respectively). Utilization of oral anticoagulant was less in patients with CAD (76.0% *vs.* 84.3%, *p* < 0.001). Concomitant use of antiplatelet was found to be a major cause of oral anticoagulant (OAC) underutilization. Specifically, the rate of OAC prescription was 95.9% in patients without antiplatelet, and 43.7% in patients with antiplatelet. Among patients with CAD who were on OAC, the rate of concomitant antiplatelet prescription was still high. In this group, 63% of patients were on triple therapy when percutaneous coronary intervention (PCI) with drug eluting stent was performed within 1 year, and 32.2% of patients without prior PCI or acute coronary syndrome were taking at least one antiplatelet with OAC.

**Conclusion:**

Among patients with concomitant CAD and AF, physicians were reluctant to discontinue antiplatelet. The use of antiplatelet discourages physicians from prescribing OAC. Underutilization of OAC may increase the risk of ischemic stroke, and an inappropriate combination of OAC and antiplatelet may increase the risk of bleeding.

*Trial registration *The trial has been registered with the Thai Clinical Trials Registry (TCTR) which complied with WHO International Clinical Trials Registry Platform dataset. The Registration Number is TCTR20160113002 (05/01/2016).

## Introduction

Coronary artery disease (CAD) and atrial fibrillation (AF) are both common cardiovascular diseases. These two conditions share common risk factors, such as older age, hypertension (HT), diabetes mellitus (DM), and smoking [1]. Therefore, coexistence of these two conditions is common in routine clinical practice.

Management of patients with concomitant CAD and AF is a challenge. Management of AF requires oral anticoagulant (OAC) for long-term prevention of stroke and systemic embolism (SSE) [1–3]. Certain spectra of CAD require one or two different antiplatelets to prevent acute coronary events [4]. When anticoagulant and antiplatelet are used together, the risk of bleeding markedly increases [5]. Recent studies focused upon and recent guidelines recommend optimized use of antithrombotic regimens in this patient population [3, 4]. In general, single antiplatelet instead of two agents along with OAC might be reasonable for patients with AF who recently received a coronary stent [6–10], CAD patients who have no recent acute coronary syndrome (ACS) or coronary interventions might be able to discontinue the use of antiplatelet if they already have an indication for OAC [11, 12]. However, current guidelines recommend selecting the antithrombotic regimen by weighing thrombotic risk against bleeding risk [13]. Some scoring systems have been proposed and validated for use in predicting bleeding in patients after coronary intervention [14, 15]. The duration of antithrombotic combination and choice of antiplatelet may influence the risk of bleeding after coronary intervention [16]. Although several thrombotic and bleeding risk scores are available in real-world clinical practice, physicians also attempt to find a balance between thrombotic risk and bleeding risk using subjective unmeasurable factors or by being more concerned about one of the two risks more than the other.

The COOL-AF Thailand registry is a national multicenter prospective cohort with the primary aim of identifying the optimal range of international normalized ratio (INR) in Thai AF patients. The objective of the present study was to investigate the characteristics and antithrombotic treatment patterns of patients with concomitant CAD and AF from Thailand’s COOL-AF registry.

## Methods

The study protocol was approved by the institutional review boards of all participating hospitals. The protocol for this registry was approved by the Central Research Ethics Committee (CREC) for Research in Human Subjects of the Ministry of Public Health, Thailand. Written informed consent was obtained from all included patients. Baseline data of patients in the COOL-AF Thailand registry were analyzed. The registry enrollment criteria were patients aged 18 years or older who were diagnosed with AF for any duration at any one of 27 public hospitals in Thailand during 2014–2017 (Thai Clinical Trials Registry study Identification Number TCTR20160113002, 05/01/2016). Key exclusion criteria were patients with rheumatic mitral stenosis, prosthetic heart valve or other moderate to severe valvular diseases, ischemic stroke within 3 months, and AF secondary to reversible causes. The primary objective of the COOF-AF registry was to identify an optimal INR for stroke prevention in Thai population diagnosed with AF. The baseline characteristics of all patients enrolled in the registry were recently published [17]. After the informed consent process, the investigators recorded data from the medical record and from patient interview into both a specially designed case record form and a web-based system. In this registry, patients were labeled as having had a history of significant CAD if patients had (1) a history of angina pectoris, (2) a history of myocardial infarction (MI) or unstable angina, (3) a history of previous percutaneous coronary intervention (PCI), or (4) a history of previous coronary artery bypass graft (CABG) surgery.

Since different spectra of CAD require a different recommended antiplatelet regimen, patients with CAD were further subclassified into (1) patients who recently (within 1 year) received a drug-eluting stent, (2) patients who received a coronary artery stent longer than 1 year previously, (3) patients who experienced acute coronary syndrome (ACS) within 1 year, but never received a stent, or (4) patients with CAD without history of ACS or receiving a stent.

According to recent guidelines, oral anticoagulant is a class I recommendation when the CHA_2_DS_2_-VASc score is 2 or more in men, and 3 or more in women. Therefore, in this article the term ‘non-gender CHA_2_DS_2_-VASc score’ was used to indicate treatment threshold (non-gender CHA_2_DS_2_-VASc score of 2 or more as class I recommendation).

The primary objective of this article was to study patterns of antithrombotic regimens among patients with AF among different spectra of coexisting CAD.

### Statistical analysis

Baseline demographic and clinical data were interpreted and described using descriptive statistics. Continuous data are presented as mean ± standard deviation, and categorical data are shown as number and percentage. Comparison was made between patients with and without CAD. Student’s *t*-test was used to compare continuous data, and chi-square test was used to compare categorical data. Univariate and multivariate logistic regression analyses were performed to identify factors significantly associated with OAC prescription. The baseline variables were compared between patients with and without OAC prescription. The variables with a *p*-value < 0.1 were included univariate and multivariate logistic regression analysis. All statistical analyses were performed using SPSS Statistics version 20 (SPSS, Inc., Chicago, IL, USA). A *p*-value < 0.05 was considered statistically significant.

## Results

Of the 3,461 patients enrolled in the COOL-AF Thailand registry, 557 (16.1%) had a history of significant CAD. Among those patients, 253 (45.4%) had a history of angina pectoris, and 283 (50.8%) had a history of MI or unstable angina. Two hundred and fifty-six (46.0%) patients underwent PCI. Among those, 42 (16.4%) patients received a drug-eluting stent (DES) within one year, and the mean duration from the date of stent implantation was 154.1 ± 96.1 days. Of those same 256 patients, 163 (63.7%) received either a DES at a time point longer than one year earlier or a bare-metal stent (BMS) at any time point. Patients had a history of prior CABG in 68 cases (12.2%) (Table 1).

**Table 1:** Clinical presentation of coronary artery disease in patients with atrial fibrillation

CAD (n = 557)	n (%)
History of angina pectoris	253 (45.4%)
History of MI/unstable angina	283 (50.8%)
Duration within 1 year	70 (24.7%)
History of PCI	256 (46.0%)
DES within 1 year	42 (16.4%)
DES longer than 1 year and/or BMS (any)	163 (63.7%)
History of CABG	68 (12.2%)
CABG within 1 year	10 (1.8%)

CAD, coronary artery disease; MI, myocardial infarction; PCI, percutaneous coronary intervention; DES, drug-eluting stent; CABG, coronary artery bypass graft surgery

When compared to patients without CAD, patients with CAD were significantly older (70.0 ± 9.9 *vs.* 66.9 ± 11.5 years, *p* < 0.001), were more likely to be men (66.6% *vs.* 56.6%, *p* < 0.001), had a higher mean CHA_2_DS_2_-VASc score (4.17 ± 1.53 *vs.* 2.78 ± 1.60, *p* < 0.001), and had a higher mean HAS-BLED score (2.01 ± 1.01 *vs.* 1.45 ± 0.98, *p* < 0.001). Even though only 2,027 from 2904 patients without CAD (69.8%) had a non-gender CHA_2_DS_2_-VASc score of 2 or more, almost all patients (537 from 557, 96.4%) with CAD had a non-gender CHA_2_DS_2_-VASc score of 2 or more. When one point was deducted from the HAD-BLED score for use of antiplatelets, that score was still significantly higher in the CAD group, but the difference between groups was markedly reduced (1.43 ± 0.90 *vs.* 1.24 ± 0.96, *p* < 0.001) (Table 2).

**Table 2:** Baseline characteristics of atrial fibrillation compared between those without and with coronary artery disease

Characteristics	CAD (n = 557)	No CAD (n = 2904)	*p*-value
Age (years)	70.0 ± 9.9	66.9 ± 11.5	** < 0.001**
Age 65–74	210 (37.7%)	902 (31.1%)	***0.002***
Age ≥ 75	185 (33.2%)	813 (28.0%)	***0.013***
Male sex	371 (66.6%)	1,643 (56.6%)	** < 0.001**
Body weight (kg)	66.3 ± 13.9	66.1 ± 14.9	0.763
BMI (kg/m^2^)	25.1 ± 4.3	25.2 ± 4.8	0.767
Type of AF			
Paroxysmal AF	188 (33.8%)	891 (30.7%)	0.152
Persistent/permanent AF	353 (63.4%)	1,938 (66.7%)	0.125
CHA_2_DS_2_VASc score	4.17 ± 1.53	2.78 ± 1.60	** < 0.001**
Non-gender CHA_2_DS_2_-VASc score	3.84 ± 1.41	2.34 ± 1.48	** < 0.001**
Non-gender CHA_2_DS_2_-VASc score 0–1	20 (3.6%)	877 (30.2%)	
Non-gender CHA_2_DS_2_-VASc score of 2 or more	537 (96.4%)	2,027 (69.8%)	
HAS-BLED score	2.01 ± 1.01	1.45 ± 0.98	** < 0.001**
Non-antiplatelet HAS-BLED score	1.43 ± 0.90	1.24 ± 0.96	** < 0.001**
Comorbidities			
History of heart failure	217 (39.0%)	706 (24.3%)	** < 0.001**
NYHA class			0.288
I	58 (26.7%)	239 (33.9%)	
II	125 (57.6%)	371 (52.5%)	
III	23 (10.6%)	56 (7.9%)	
IV	3 (1.4%)	9 (1.3%)	
LVEF (%)	54.4 ± 17.1	61.0 ± 14.0	** < 0.001**
Hypertension	416 (74.7%)	1,948 (67.1%)	** < 0.001**
Diabetes mellitus	205 (36.8%)	645 (22.2%)	** < 0.001**
History of TIA/stroke	81 (14.5%)	521 (17.9%)	0.053
PAD	17 (3.1%)	28 (1.0%)	** < 0.001**
History of major bleeding	15 (22.7%)	57 (21.8%)	0.865
Intracranial hemorrhage	1 (6.7%)	18 (31.6%)	0.096
GI hemorrhage	11 (73.3%)	26 (45.6%)	0.056
Chronic kidney disease			
eGFR (mL/min)	48.5 ± 20.0	57.4 ± 21.2	** < 0.001**
GFR < 60 mL/min	333 (73.7%)	1,254 (59.0%)	** < 0.001**
On renal replacement therapy	17 (3.1%)	23 (0.8%)	** < 0.001**
Chronic liver disease	13 (2.3%)	68 (2.3%)	0.991
Labile INR	171 (30.7%)	724 (24.9%)	***0.004***
CIED	82 (14.7%)	263 (9.1%)	** < 0.001**
Pacemaker	51 (62.2%)	222 (84.4%)	** < 0.001**
ICD/CRT-D	31 (37.8%)	40 (15.2%)	** < 0.001**
CRT-P/CRT-D	9 (11.0%)	8 (3.0%)	***0.007***

Data presented as mean ± standard deviation or number and percentage

A *p*-value < 0.05 indicates statistical significance

CAD, coronary artery disease; BMI, body mass index; AF, atrial fibrillation; HAS-BLED, Hypertension, Abnormal liver/renal function, Stroke history, Bleeding history or predisposition, Labile INR, Elderly, Drug/alcohol usage; NYHA, New York Heart Association; LVEF, left ventricular ejection fraction; TIA, transient ischemic attack; PAD, peripheral arterial disease; GI, gastrointestinal; eGFR, estimated glomerular filtration rate; INR, international normalized ratio; CIED, cardiovascular implantable electronic devices; ICD, implantable cardioverter defibrillator; CRT-D, cardiac resynchronization therapy defibrillator; CRT-P, cardiac resynchronization therapy pacemaker

CAD patients had more comorbidities than non-CAD patients, and this was reflected in a higher CHA_2_DS_2_-VASc score among CAD patients. The CAD group had more heart failure (HF) (39.0% *vs.* 24.3%, *p* < 0.001), more HT (74.7% *vs.* 67.1%, *p* < 0.001), more DM (36.8% *vs.* 22.2%, *p* < 0.001), more peripheral artery disease (PAD) (3.1% *vs.* 1.0%, *p* < 0.001), more moderate to severe chronic kidney disease (CKD) (65.7% *vs.* 51.2% *p* < 0.001), and they had more implantation of a cardiovascular implantable electronic device (CIED) (14.7% *vs.* 9.1%, *p* < 0.001). However, history of stroke and/or transient ischemic attack (TIA) (14.5% *vs.* 17.9%, *p* = 0.053) and prior bleeding (22.7% *vs.* 21.8%, *p* = 0.865) were not significantly different between the CAD and non-CAD groups.

Among all patients enrolled in the registry, patients with CAD ware taking more antiplatelets than patients without CAD (57.1% *vs.* 20.3%, *p* < 0.001). However, the rate of OAC use was not significantly different between groups (75.9% *vs.* 75.2%, *p* = 0.699). The majority of patients in both groups used warfarin as an OAC (91.0% *vs.* 91.2%, *p* > 0.05) (Table 3).

**Table 3:** Baseline antithrombotic therapy and cardiovascular medications

Therapy and medications	CAD (n = 557)	No CAD (n = 2,904)	*p*-value
*Antiplatelet use, n (%)*	318 (57.1%)	589 (20.3%)	** < 0.001**
Single antiplatelet	245 (77.0%)	560 (95.1%)	
ASA	195 (79.6%)	506 (90.4%)	
P2Y12 inhibitors	50 (20.4%)	54 (9.6%)	
Dual antiplatelet	73 (23.0%)	26 (4.4%)	
*Oral anticoagulants, n (%)*	423 (75.9%)	2,183 (75.2%)	0.699
Warfarin	385 (91.0%)	1,991 (91.2%)	
NOACs	38 (9.0%)	192 (8.8%)	
Rate and rhythm control medications, n (%)			
Beta blocker	430 (77.2%)	1,994 (68.7%)	** < 0.001**
Diltiazem	11 (2.0%)	72 (2.5%)	0.476
Verapamil	4 (0.7%)	29 (1.0%)	0.533
Digoxin	66 (11.8%)	477 (16.4%)	***0.007***
Amiodarone	40 (7.2%)	259 (8.9%)	0.181
Other AAD	5 (0.9%)	79 (2.7%)	***0.010***
Other medications, n (%)			
DHP-CCB	130 (23.3%)	727 (25.0%)	0.396
PPI	194 (34.8%)	523 (18.0%)	** < 0.001**
Statin	471 (84.6%)	1,572 (54.1%)	** < 0.001**
ACEI	158 (28.4%)	616 (21.2%)	** < 0.001**
ARB	172 (30.9%)	648 (22.3%)	** < 0.001**

Data presented as number and percentage

A *p*-value < 0.05 indicates statistical significance

CAD, coronary artery disease; ASA, aspirin; NOACs, non-vitamin K antagonist oral anticoagulants; AAD, antiarrhythmic drug; DHP-CCB, dihydropyridine calcium channel blocker; PPI, proton pump inhibitor; ACEI, angiotensin-converting enzyme inhibitor; ARB, angiotensin receptor blocker

### OAC prescription in patients with and without CAD

**Fig. 1:**
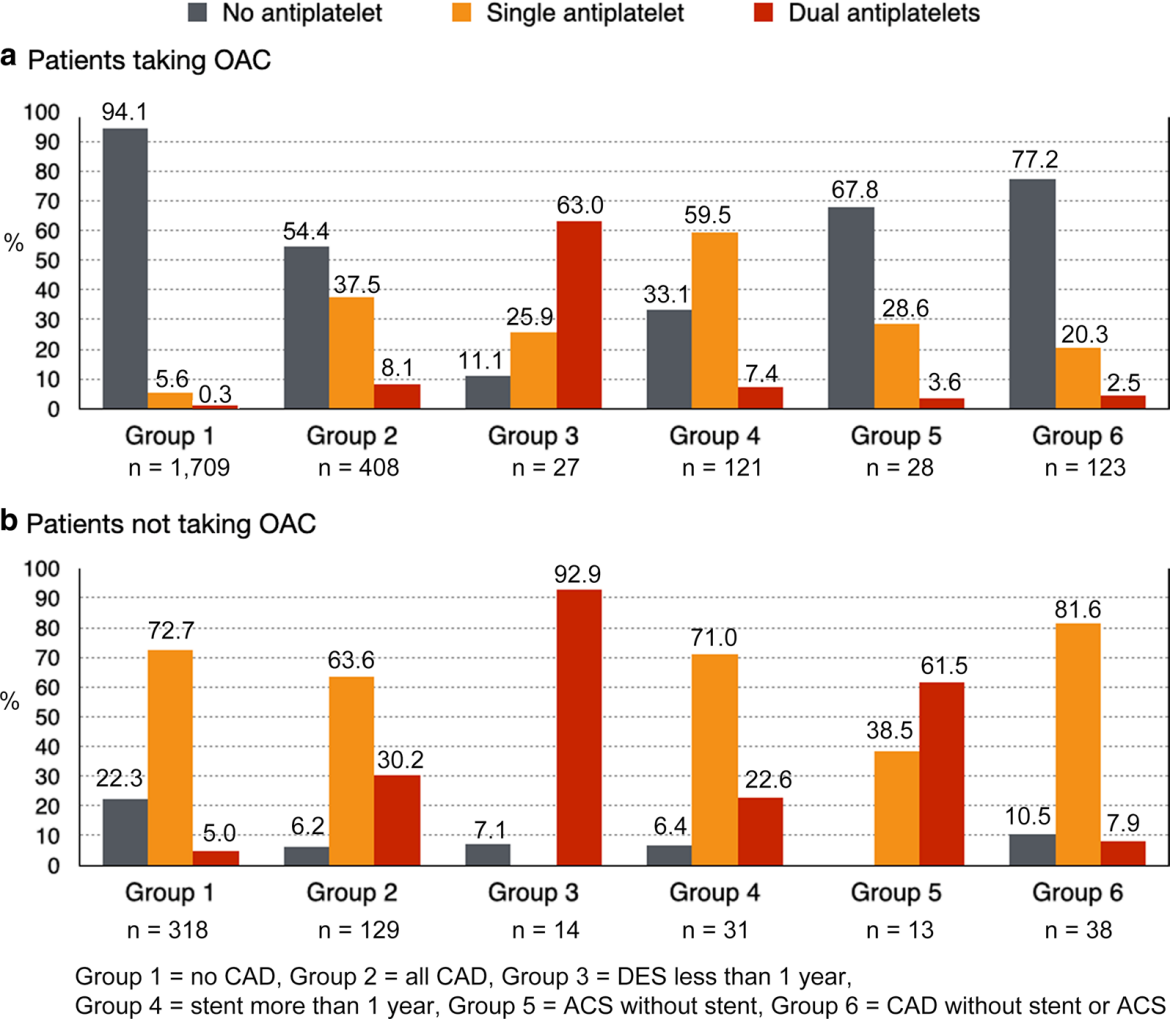
Demonstrates oral anticoagulant (OAC) and antiplatelet prescription pattern among different groups of patients with atrial fibrillation and a non-gender CHA_2_DS_2_-VASc score of 2 or more. Group 1 = patients without history of coronary artery disease (CAD); Group 2 = patients with history of coronary artery disease (overall); Group 3 = patients who underwent percutaneous coronary intervention (PCI) with drug-eluting stent (DES) within 1 year; Group 4 = patients who underwent percutaneous coronary intervention with stent longer than 1 year; Group 5 = patients who experienced acute coronary syndrome (ACS) without receiving a stent; and, Group 6 = patients with coronary artery disease without history of acute coronary syndrome or receiving a stent

Although use of OAC was not significantly different among overall patients compared between those with and without CAD, OAC use in patients with CAD who had more indications for OAC (patients with non-gender CHA_2_DS_2_-VASc score of 2 or more) was significantly lower than in patients without CAD who had indication(s) for OAC (76.0% *vs.* 84.3%, *p* < 0.001). This finding was observed in all CAD subpopulation, including patients who never had ACS or who never received any stent (76.4% in patients with CAD who never had ACS or stent *vs*. 84.3% in patients without CAD, *p* = 0.009). Use of OAC was lowest in patients who received a DES within 1 year (65.9%) (Fig. 1 and Table 4).

**Table 4:** Statistical analysis of data from Fig. 1

	Gr 1 versus Gr 2 (*p*-value)	Gr 1 versus Gr 6 (*p*-value)	Gr 3 versus Gr 4 (*p*-value)	Gr 3 versus Gr 6 (*p*-value)	Gr 4 versus Gr 6 (*p*-value)	Gr 5 versus Gr 6 (*p*-value)
Rate of OAC	84.3 *vs.* 76.0 ** < 0.001**	84.3 *vs.* 76.4 ***0.009***	65.9 *vs.* 79.6 0.065	65.9 *vs.* 76.4 0.168	79.6 *vs.* 76.4 0.494	68.3 *vs.* 76.4 0.286
No OAC						
No AP	22.3 *vs.* 6.2 ** < 0.001**	22.3 *vs.* 10.5 0.092	7.1 *vs.* 6.5 0.931	7.1 *vs.* 10.5 0.714	6.5 *vs.* 10.5 0.684	–-
1 AP	72.3 *vs.* 63.6 ** < 0.001**	72.3 *vs.* 81.6 0.223	–-	–-	71.0 *vs.* 81.6 0.299	38.5 *vs.* 81.6 ***0.011***
2 AP	5.0 *vs.* 30.2 ** < 0.001**	5.0 *vs.* 7.9 0.441	92.9 *vs.* 22.6 ** < 0.001**	92.9 *vs.* 7.9 ** < 0.001**	22.6 *vs.* 7.9 0.100	61.5 *vs.* 7.9 ** < 0.001**
Taking OAC						
No AP	94.1 *vs.* 54.4 ** < 0.001**	94.1 *vs.* 77.2 ** < 0.001**	11.1 *vs.* 33.1 ***0.023***	11.1 *vs.* 77.2 ** < 0.001**	33.1 *vs.* 77.2 ** < 0.001**	67.9 *vs.* 77.2 0.298
1 AP	5.5 *vs.* 37.5 ** < 0.001**	5.5 *vs.* 20.3 ** < 0.001**	25.9 *vs.* 59.5 ***0.002***	25.9 *vs.* 20.3 0.520	59.5 *vs.* 20.3 ** < 0.001**	28.6 *vs.* 20.3 0.341
2 AP	0.3 *vs.* 8.1 ** < 0.001**	0.3 *vs.* 4.3 ***0.013***	63.0 *vs.* 7.4 ** < 0.001**	63.0 *vs.* 4.3 ** < 0.001**	7.4 *vs.* 4.3 0.071	3.6 *vs.* 4.3 0.564

A *p*-value < 0.05 indicates statistical significance

OAC, oral anticoagulant; AP, antiplatelet

Group 1 = patients without history of coronary artery disease

Group 2 = patients with history of coronary artery disease (overall)

Group 3 = patients who underwent percutaneous coronary intervention with drug-eluting stent within 1 year

Group 4 = patients who underwent percutaneous coronary intervention with stent longer than 1 year

Group 5 = patients who experienced acute coronary syndrome without receiving a stent

Group 6 = patients with coronary artery disease without history of acute coronary syndrome or receiving a stent

Univariate and multivariate analysis for factors significantly and/or independently associated with OAC prescription is shown in Table 5. The variables in the table were selected from baseline variables (in Table 2) that had p-value < 0.1 for the difference between patients with and without OAC prescription. Concurrent antiplatelet therapy showed the strongest association with lower use of OAC. In 2564 patients who had a non-gender CHA_2_DS_2_-VASc score of 2 or more with or without CAD, 1830 of 1909 (95.9%) patients who took no antiplatelet were taking OAC. In contrast, only 285 of 552 patients (43.7%) taking at least one antiplatelet were taking OAC (adjusted OR: 0.02, 95% CI 0.01–0.03, *p* < 0.001). Patients taking a single antiplatelet and two antiplatelets were taking OAC in 44.2% and 40.9% of patients, respectively.

**Table 5:** Univariate and multivariate analysis for factors significantly and independently associated with anticoagulant prescription

Factors	Univariate analysis		Multivariate analysis	
	OR (95% CI)	*p*	OR (95% CI)	*p*
BMI > 25 kg/m^2^	1.23 (0.99–1.52)	0.065		
Paroxysmal AF versus permanent AF	0.68 (0.55–0.84)	** < 0.001**	0.74 (0.55–1.00)	***0.048***
CAD	0.59 (0.47–0.74)	** < 0.001**	2.49 (1.76–3.52)	** < 0.001**
Heart failure	0.72 (0.58–0.89)	***0.003***		
Ischemic stroke or TIA	2.54 (1.88–3.43)	** < 0.001**	1.85 (1.25–2.74)	***0.002***
PAD	0.54 (0.28–1.06)	0.072		
On renal replacement therapy	0.31 (0.16–0.61)	***0.001***		
Chronic liver disease	0.48 (0.23–1.02)	0.055		
Antiplatelet use	0.03 (0.03–0.04)	** < 0.001**	0.02 (0.01–0.03)	** < 0.001**
Rate and rhythm control medications				
Beta-blocker	1.34 (1.08–1.66)	***0.008***	1.81 (1.32–2.46)	** < 0.001**
Proton pump inhibitors	0.54 (0.43–0.67)	** < 0.001**		
Statin	1.29 (1.04–1.59)	***0.020***	1.97 (1.43–2.72)	** < 0.001**
ACEI	1.34 (1.04–1.72)	***0.024***		
Diuretic	1.22 (0.98–1.52)	0.076		

A *p*-value < 0.05 indicates statistical significance

OR, odds ratio; CI, confidence interval; BMI, body mass index; AF, atrial fibrillation; CAD, coronary artery disease; TIA, transient ischemic attack; PAD, peripheral arterial disease; ACEI, angiotensin-converting enzyme inhibitor

Although CAD was found to be associated with lower use of OAC. However, after adjusting for other factors (especially antiplatelet status), CAD was instead associated with more use of OAC. Another factor independently associated with lower use of OAC was paroxysmal AF (against permanent AF) (adjusted OR: 0.74, 95% CI 0.55–1.00, *p* = 0.048). Factors independently associated with more use of OAC included history of ischemic stroke or TIA (adjusted OR: 1.85, 95% CI 1.25–2.74, *p* = 0.002), use of beta-blocker (adjusted OR: 1.81 95% CI 1.32–2.46, *p* < 0.001), and use of statin (adjusted OR: 1.97, 95% CI 1.43–2.72, *p* < 0.001) (Table 5).

### Additional antiplatelet in patients already taking OAC

In patients without CAD, an antiplatelet prescription on top of OAC was uncommon (5.5% for single antiplatelet, and 0.3% for two antiplatelets). In contrast, up to 37.5% of patients with any spectra of CAD were taking single antiplatelet, and 8.1% were taking two antiplatelets [significantly higher than in patients without CAD (*p* < 0.001)]. Addition of antiplatelet was even observed in CAD patients without history of ACS or prior PCI with stent (20.3% *vs.* 5.5%, *p* < 0.001 for single antiplatelet, and 4.3% *vs.* 0.3%, *p* = 0.013 for two antiplatelets)(Fig. 1 and Table 4).

Among CAD patients who received a DES within the preceding one year, additional antiplatelet regimens were significantly more aggressive than in CAD patients with no history of ACS or PCI with stent [63% *vs.* 4.3% (*p* < 0.001) were taking concomitant dual antiplatelets (so-called triple antithrombotic therapy—TAT), and 25.9% were taking concomitant single antiplatelet].

After 1 year of PCI, prescription of TAT was significantly reduced (63.0 *vs.* 7.4, *p* < 0.001) to a proportion similar to that of CAD patients who never had PCI or ACS (7.4% *vs.* 4.3%, *p* = 0.071). However, a considerable number of patients after 1 year of PCI maintained single antiplatelet addition to OAC when compared to CAD patients who never had PCI or ACS (59.5% *vs.* 20.3%, *p* < 0.001).

Antiplatelet prescription practice was not significantly different between CAD patients who experienced ACS without undergoing PCI and CAD patients who did experience ACS with no PCI (28.6% *vs.* 20.3%, *p* = 0.341 for single antiplatelet therapy, and 3.6% *vs.* 4.3%, *p* = 0.564 for dual antiplatelet therapy).

**Fig. 2:**
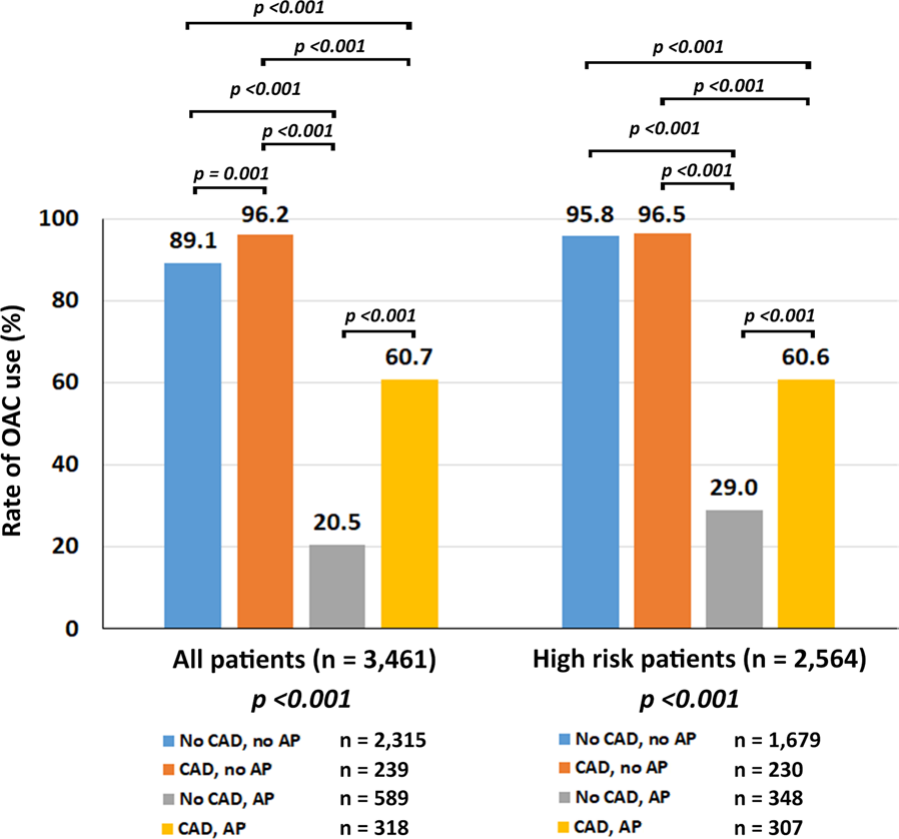
Rate of oral anticoagulant (OAC) use among 4 groups of patients according to the presence of coronary artery disease (CAD) and the use of antiplatelet (AP) for all patients (left) and high-risk patients (right). High-risk patients defined as male with CHA2DS2-VASc score ≥ 2 or female with CHA2DS2-VASc score ≥ 3

The rate of OAC use was analyzed according to CAD and antiplatelet status in all patients and in high-risk group defined as CHA2DS2VASc ≥ 2 in men and ≥ 3 in women. The proportion of CAD patients in high-risk group was greater than non-high-risk group [537 (20.9%) vs 20 (2.2%), p < 0.001]. The proportion of antiplatelet use was not difference between high-risk and non-high-risk group [655 (25.5%) vs 252 (28.1%), p = 0.135]. The results of rate of OAC use in all patients and in high-risk group according to CAD and antiplatelet use are shown in Fig. 2. There was a significant interaction of the presence of CAD, and the use of antiplatelet on the rate of OAC use (p-value for interaction < 0.001). The rate of OAC use is low in patients who use antiplatelet (Fig. 2). Patients with CAD without antiplatelet use had an OR and 95% CI of OAC use of 3.13 (1.59–6.25) (p = 0.001) compared to patients without CAD and no antiplatelet. However, patients with CAD and antiplatelet use had an OR and 95% CI of OAC use of 0.19 (0.15–0.25) (p < 0.001) compared to those without CAD and no antiplatelet. This relation persisted after the adjustment of confounders.

### High risk versus low risk of bleeding according to HAS-BLED score

Patients with CAD with a CHA_2_DS_2_-VASc score of 2 or more who were taking OAC were further classified by bleeding risk into high risk (3 or more points) or low risk (0–2 points) according to their HAS-BLED score. In this setting, a point for use of antiplatelet was deducted from their HAS-BLED score to improve the physician’s decision whether or not to prescribe antiplatelet. The results showed no significant difference in antiplatelet prescription between the high and low bleeding risk groups (Table 6).

**Table 6:** Antiplatelet prescription in patients already using OAC classified by HAS-BLED score

	Total patients	Non-antiplatelet HAS-BLED score		*p*
		0–2	3 or more	
All CAD, n (%)	408	353	55	
No antiplatelet	222 (54.4%)	196 (55.5%)	26 (47.3%)	0.253
Single antiplatelet	153 (37.5%)	128 (36.3%)	25 (45.5%)	0.190
Dual antiplatelets	33 (8.1%)	29 (8.2%)	4 (7.3%)	1.000
DES < 1 year, n (%)	27	23	4	
No antiplatelet	3 (11.1%)	3 (13.0%)	0 (0.0%)	1.000
Single antiplatelet	7 (25.9%)	6 (26.1%)	1 (25.0%)	1.000
Dual antiplatelets	17 (63.0%)	14 (60.9%)	3 (75.0%)	1.000
DES > 1 year or BMS for any duration, n (%)	125	108	17	
No antiplatelet	41 (32.8%)	35 (32.4%)	6 (35.3%)	0.814
Single antiplatelet	73 (58.4%)	63 (58.3%)	10 (58.8%)	0.970
Dual antiplatelets	11 (8.8%)	10 (9.3%)	1 (5.9%)	1.000
History of ACS within 1 year, n (%)	42	33	9	
No antiplatelet	21 (50.0%)	17 (51.5%)	4 (44.4%)	1.000
Single antiplatelet	12 (28.6%)	9 (27.3%)	3 (33.3%)	0.699
Dual antiplatelets	9 (21.4%)	7 (21.2%)	2 (22.2%)	1.000
CAD without prior ACS or PCI, n (%)	386	332	54	
No antiplatelet		189 (56.9%)	26 (48.1%)	0.102
Single antiplatelet		115 (34.6%)	25 (46.3%)	0.053
Dual antiplatelets		28 (8.4%)	3 (5.6%)	1.000

A *p*-value < 0.05 indicates statistical significance

OAC, oral anticoagulant; HAS-BLED, Hypertension, Abnormal liver/renal function, Stroke history, Bleeding history or predisposition, Labile INR, Elderly, Drug/alcohol usage; CAD, coronary artery disease; DES, drug-eluting stent; BMS, bare-metal stent; ACS, acute coronary syndrome; PCI, percutaneous coronary intervention

## Discussion

CAD is a common comorbidity in patients with AF. The prevalence of CAD in AF patients ranged from 16.6 to 36.5% according to the definition of CAD [18–21]. The results of this study showed a prevalence of CAD of 16.1% in Thai patients with AF. Patients with CAD were older and had more other comorbidities, including HT, DM, HF, PAD, and CKD. Therefore, in addition to CAD patients being at greater risk for stroke and systemic embolism, they also have more comorbidities than AF patients without CAD. As a result, almost all patients with coexisting AF and CAD had a non-gender CHA_2_DS_2_-VASc score of 2 or more, and they had an indication for OAC (96.4%).

Patients with concomitant CAD tend to have a lower time in therapeutic range (TTR). The results of this study showed that TTR was 49.7 ± 28.3 in CAD compared to 52.5 ± 27.3 for no CAD (p = 0.077). Explanations for this finding may include: (1) CAD patients tended to have more comorbidities, which is a predictor of poor INR control, (2) patients with CAD received more proton pump inhibitor, which interacts with warfarin, and (3) physicians might attempt to maintain a lower and narrower INR range (e.g., 2.0–2.5) when antiplatelet is co-administered.

The presence of any spectra of CAD associated with less OAC prescription. The results of this study found that only 76% of patients with CAD received OAC compared to 84.3% of patients without CAD when OAC was indicated. Utilization of OAC was lowest (65.9%) in patients who received a DES within the previous 1 year. In fact, CAD itself associated with more use of OAC according to result of the multivariate analysis because it is one of the risk factors included in the CHA_2_DS_2_-VASc score. However, use of antiplatelet was found to be the strongest factor discouraging physicians from prescribing OAC, and CAD patients are associated with use of antiplatelets.

In this study, the use of dual antiplatelet therapy on top OAC (so-called triple antithrombotic therapy) in patients who underwent PCI with DES within the previous 1 year was very common (63.0%). By and during the registry’s enrollment period, several studies had already been published that reported data that support the use of only one antiplatelet on top OAC in patients with AF who underwent recent PCI. The What is the Optimal antiplatElet and anticoagulant therapy in patients with oral anticoagulation and coronary StenTing (WOEST) study was an open-label, randomized controlled study that included 573 patients who underwent PCI and who were taking oral anticoagulant. Use of clopidogrel without aspirin caused 64% less bleeding events, and no significant increase in the rate of thrombotic episodes [6]. A meta-analysis of RCTs and adjusted observational studies that included 4,318 patients with various indications for OAC showed 21.0% less bleeding with no increase in combined death, MI, stroke, or stent thrombosis [22]. More recent studies in NOACs showed similar results [7–10]. However, taking DAPT without OAC led to 44.0% more thromboembolic events in patients with AF when compared to AF taking OAC alone [23]. Despite this growing evidence, the prescription pattern from this study indicates that physicians tended to give priority to dual antiplatelet therapy rather than oral anticoagulant. This may result in increased use of triple therapy, which can increase bleeding risk, and increased use of DAPT without OAC, which can increase the risk of stroke.

Recent guidelines also encourage physicians to prescribe OAC without additional antiplatelet in patients who underwent PCI with stent earlier than one year previously. Physicians in this study were more likely to continue at least one antiplatelet together with OAC in more than a half of this subpopulation. The Optimizing Antithrombotic Care in Patients with Atrial Fibrillation and Coronary Stent (OAC-ALONE) study was an open-label, non-inferiority study that compared OAC alone to OAC with antiplatelet. Three-quarters of the study population were taking warfarin, and the rest were taking NOACs. That study was underpowered by premature termination of enrollment and failed to prove non-inferiority [24]. The recently published Atrial Fibrillation and Ischemic events with Rivaroxaban in patiEnts with stable coronary artery disease (AFIRE) study was an another open-label study that compared rivaroxaban 15 or 10 mg alone to rivaroxaban 15 or 10 mg plus one antiplatelet agent. That study included 2236 patients who had history of either CABG or PCI within the previous 1 year. Rivaroxaban monotherapy was found to be superior to combination therapy for both the primary efficacy endpoint (composite of stroke, systemic embolism, MI, unstable angina requiring revascularization, or death from any cause) and the primary safety endpoint (major bleeding) [12]. More data may be needed to convince physicians to discontinue antiplatelet in this scenario.

The HAS-BLED score was introduced in the European Society of Cardiology (ESC) guideline in 2010 [25]. A high HAS-BLED score itself is not a contraindication for OAC, but it helps physicians to focus on modifiable bleeding risk, including unnecessary addition of antiplatelet. More recent guidelines and consensus opinions use the HAS-BLED score to help individualize antithrombotic regimen when concomitant use of antiplatelet is needed. The results of this study showed no significant association between HAS-BLED score and choice of antithrombotic regimen. This finding suggests that physicians may be more concerned about stent complications than bleeding risk. However, there might be unmeasurable patient characteristics that contribute to a physician’s decision to discontinue antiplatelet when OAC is needed.

Based on the results of this study, future research should focus on the use of OAC and antiplatelet according to the standard practice guidelines [3, 13, 26]. In stable CAD patients with AF, OAC should be used without antiplatelet. However, in AF patients with recent ACS or PCI, triple therapy should be used for a short duration followed by OAC plus single antiplatelet for up to 12 months.

### Limitations

First, although the COOL-AF Thailand was a prospective cohort, for this study, patient baseline characteristics were collected from data in the medical record and from patient interview. As such, there is a small, but possible chance of some missing data. Second, details specific to the placement of drug-eluting stents and the complexity of coronary lesions in patients who underwent PCI were not collected. Third, antithrombotic regimen pattern data were collected at a time of enrollment. However, the exact duration of each antithrombotic regimen after ACS or PCI could not be assessed. It is also possible that the regimen was more aggressive before the time of enrollment. Lastly, the majority of OAC in this study was warfarin. The major reason for the high rate of warfarin use in the current study is that warfarin is a much more affordable medication compared more contemporary and expensive medications like NOACs. In Thailand, warfarin is promoted as the first choice of OAC among the national healthcare coverage schemes. To use NOACs, physicians need to submit a Drug Utilization Evaluation (DUE) form with a good reason for their use in that patient.

## Conclusion

Utilization of oral anticoagulant was less in patients with CAD compared to those without CAD. Use of antiplatelet is the strongest factor associated with non-prescription of OAC. A significant proportion of patients received antiplatelet combined with OAC without indication. Under use of OAC may increase the risk of ischemic stroke, and the inappropriate combination of OAC and antiplatelet may increase the risk of bleeding.

## Data Availability

The dataset that was used to support the conclusion of this study is included within the manuscript. Any other additional data will be made available upon request to Rungroj Krittayaphong (rungroj.kri@mahidol.ac.th).

## Abbreviations

AAD: Antiarrhythmic drug
ACEI: Angiotensin-converting enzyme inhibitor
AF: Atrial fibrillation
ARB: Angiotensin receptor blocker
ASA: Aspirin
BMI: Body mass index
CABG: Coronary artery bypass graft surgery
CAD: Coronary artery disease
CIED: Cardiovascular implantable electronic device
CRT-D: Cardiac resynchronization therapy defibrillator
CRT-P: Cardiac resynchronization therapy pacemaker
DES: Drug-eluting stent
DHP-CCB: Dihydropyridine calcium channel blocker
eGFR: Estimated glomerular filtration rate
GI: Gastrointestinal
ICD: Implantable cardioverter defibrillator
INR: International normalized ratio
MI: Myocardial infarction
NOACs: Non-vitamin K antagonist oral anticoagulants
NYHA: New York Heart Association
PAD: Peripheral arterial disease
PCI: Percutaneous coronary intervention
PPI: Proton pump inhibitor
SD: Standard deviation
TIA: Transient ischemic attack
